# Long-term tonic spinal cord stimulation in advanced Parkinson’s disease: No effect from stimulation under placebo-controlled evaluation

**DOI:** 10.1016/j.prdoa.2023.100220

**Published:** 2023-10-06

**Authors:** Rafael Bernhart Carra, Tamine Teixeira da Costa Capato, Janaina Reis Menezes, Egberto Reis Barbosa, Kleber Paiva Duarte, Manoel Jacobsen Teixeira, Rubens Gisbert Cury

**Affiliations:** a Movement Disorders Center, Department of Neurology, School of Medicine, University of São Paulo, São Paulo, Brazil; b Functional Neurosurgery Division, Department of Neurology, School of Medicine, University of São Paulo, São Paulo, Brazil

**Keywords:** Spinal Cord Stimulation, Parkinson’s Disease, Freezing of Gait

## Abstract

• Chronic spinal cord stimulation effectiveness was evaluated in four PD patients.
• Double blinded cross over evaluation was performed using subthreshold stimulation.
• An open label evaluation with regular suprathreshold stimulation was also performed.
• No statistically significant effect was produced with either stimulation.
• This study highlights the lack of strong clinical evidence supporting SCS for PD.

Spinal cord stimulation (SCS) for Parkinson’s disease (PD) has been proposed as a possible treatment for PD patients with gait impairment, and is corroborated by a recent meta-analysis [1], despite only a single double blinded placebo-controlled trial published [2] in 11 years of study. We documented good initial results in a previous unblinded study in our center [3]; however, over time, the perception of benefit among our patients declined. As long-term reports are also rare and lacking controlled evaluations [4], we set out to study long-term SCS effectiveness through a randomized double-blinded sham-controlled crossover evaluation.

Patients with more than three years of SCS still in follow-up were selected and randomly assigned to either subthreshold tonic stimulation or sham stimulation by an uninvolved researcher, evaluated after completing four weeks of continuous stimulation, and crossed over, with examiners and patients blinded to settings. For blinding, the stimulation amplitude in subthreshold settings was 90 % of the minimal paresthesia-inducing threshold in the supine position. After the cross over evaluations a suprathreshold phase was conducted with stimulation set to regular perceivable amplitudes for another four weeks before a final unblinded evaluation. Stimulation parameters other than amplitude remained unchanged from the last regular follow-up session. Evaluation was performed on the ON medication status at the same time of the day and consisted of the Timed Up and Go (TUG) test, Unified Parkinson’s Scale Part III (UPDRS part III), Parkinson’s Disease Questionnaire 39 (PDQ-39), freezing of gait questionnaire and new freezing of gait questionnaire (FOG-Q and NFOG-Q, respectively), fall efficacy scale international (FES-I), and visual analog scale (VAS) for pain.

A total of nine PD patients were subjected to SCS in our center three years or more before this trial; all were implanted with paddle electrodes (Medtronic Inc) at T2-T4 level, all originally selected for refractory gait complaints with or without freezing of gait (FoG). Three patients were lost to follow up, one had an early device malfunction and chose not to undergo a new procedure due to no perception of benefit, and another died due to a cardiac event shortly before completing six months of therapy. The four remaining patients were recruited for this trial, with age from 56 to 73 years and PD duration between 12 and 28 years. Patient one was the single female, and completed 7 years of SCS, whereas the others had just completed 3 years of therapy. Patients one and three also complained of pain at the last follow-up, and all except patient four used subthalamic nucleus deep brain stimulation devices, stimulation parameters were unchanged during this trial. Only patients one and four reported perception of benefit from SCS. All patients completed all trial phases, although patient two only completed the open suprathreshold phase after a 4-month delay. See supplementary files for additional information on trial design, patients, stimulation settings and outcomes.

There was no statistically significant effect from subthreshold or suprathreshold stimulation when compared to sham stimulation (Fig. 1). Pain was slightly reduced in patients one and three in both subthreshold and suprathreshold stimulation phases, but this did not seem to have provided an expected increase in mobility or quality of life from pain control. Suprathreshold stimulation while not statistically significant led to numerically worse outcomes than subthreshold stimulation, which could be a result of the increased anticipation after two months of paresthesia-free settings or even disappointment due to the lack of noticeable change after returning to paresthesia-inducing settings, or dissemination of current to motor sections of the spinal cord, or chance.

**Fig. 1:**
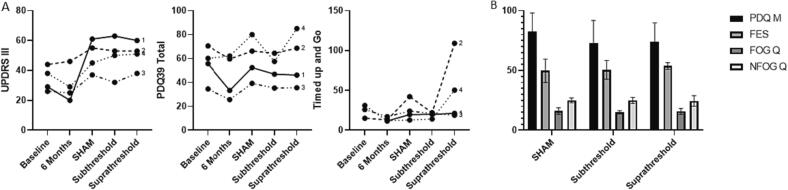
A. Longitudinal evaluation of individual UPDRS III and PDQ 39 scores and timed up-and-go times on regular medication. B. Outcomes limited to double blinded randomized evaluations (SHAM and subthreshold) and subsequent unblinded suprathreshold evaluation.

This is the second randomized double-blind sham-controlled trial in SCS for PD and as the first produced negative results, findings at odds with the results from most case reports and series. Optimistically it is possible that either the patients were non-responders or that stimulation parameters were inadequate, despite adjustments over the entire follow-up. Subthreshold stimulation does not seem to be responsible for the lack of findings as suprathreshold stimulation was also not effective, a finding also documented in the previous double blinded study [2] and a more recent longitudinal study [5]. Finally, while our population is highly heterogeneous it is not substantially different from most patients subjected to SCS, with advanced PD and gait disorders [1].

It’s however much more worrying that SCS might simply not be effective and perhaps rely on placebo effect, with early improvement mainly dependent on placebo effects as also suggested previously [5], as most trials to date have been unblinded due to regular suprathreshold tonic stimulation being perceivable. Our results should be read as a warning against clinical decision making based on unblinded trials and greatly highlight the need for further adequate controlled trials, especially considering that unperceivable BURST stimulation or subthreshold tonic stimulation currently enable placebo control.

## 1 Author roles

1. Research project: A. Conception, B. Organization, C. Execution;
2. Statistical Analysis: A. Design, B. Execution, C. Review and Critique;
3. Manuscript Preparation: A. Writing of the first draft, B. Review and Critique;

RBC: 1ABC, 2ABC, 3A

TTCC 1.ABC, 3A

JRM 1.ABC, 3A

ERB 1.AB, 3B

KPD 1C, 3B

MJT 1B, 3B

RGC: 1AB,2C,3C

## 2 Funding Sources

This research received funding from São Paulo Research Foundation (FAPESP), grant #2019/11000-8.

## 3 Financial Disclosures for the previous 12 months

The authors declare that there are no additional disclosures to report.

## 4 Statement of Ethics

Study approval statement: This study was reviewed and approved by the Institutional Review Board (CAPPESQ-HCFMUSP #12690213.0.0000.0068).

Consent to participate statement: All participants provided written informed consent before implementing any study protocol.

We confirm that we have read the Journal’s position on issues involved in ethical publication and affirm that this work is consistent with those guidelines.

## Declaration of Competing Interest

The authors declare the following financial interests/personal relationships which may be considered as potential competing interests: Rubens Gisbert Cury reports financial support was provided by São Paulo Research Foundation (FAPESP).

## References

[1] C. Sarica, A. Zemmar, O. Yousefi, A.C. Yang, A. Uzuner, Z. Sheng, et al. Spinal Cord Stimulation for Parkinson’s Disease: A Systematic Review and Meta-Analysis of Pain and Motor Outcomes. Stereotactic and Functional Neurosurgery. July 2023;1. 10.1159/000531089. 10.1159/000531089 PMC10614495 37429256

[2] Thevathasan W., Mazzone P., Jha A., Djamshidian A., Dileone M., Di Lazzaro V., Brown P. (2010). Spinal cord stimulation failed to relieve akinesia or restore locomotion in Parkinson disease. Neurology.

[3] Pinto de Souza C., Hamani C., Oliveira Souza C., Lopez Contreras W.O., Dos Santos Ghilardi M.G., Cury R.G. (2017). Spinal cord stimulation improves gait in patients with Parkinson's disease previously treated with deep brain stimulation. Mov. Disord..

[4] O. Samotus, A. Parrent, M. Jog, Long-term update of the effect of spinal cord stimulation in advanced Parkinson's disease patients. Brain Stimul. 2020 Sep-Oct;13(5):1196-1197. 10.1016/j.brs.2020.06.004. 10.1016/j.brs.2020.06.004 32504828

[5] Prasad S., Aguirre-Padilla D.H., Poon Y.Y., Kalsi-Ryan S., Lozano A.M., Fasano A. (2020). Spinal Cord Stimulation for Very Advanced Parkinson's Disease: A 1-Year Prospective Trial. Mov. Disord..

