# Supplementary material for: Long-term tonic spinal cord stimulation in advanced Parkinson’s disease: No effect from stimulation under placebo-controlled evaluation
Source: Clin Park Relat Disord. 2023 Oct 6;9:100220. doi: 10.1016/j.prdoa.2023.100220 (PMC10580045; doi:10.1016/j.prdoa.2023.100220)
Supplement: Supplementary Data 1 [file mmc1.docx]

Supplementary Files:

Supplementary figure 1. Study design


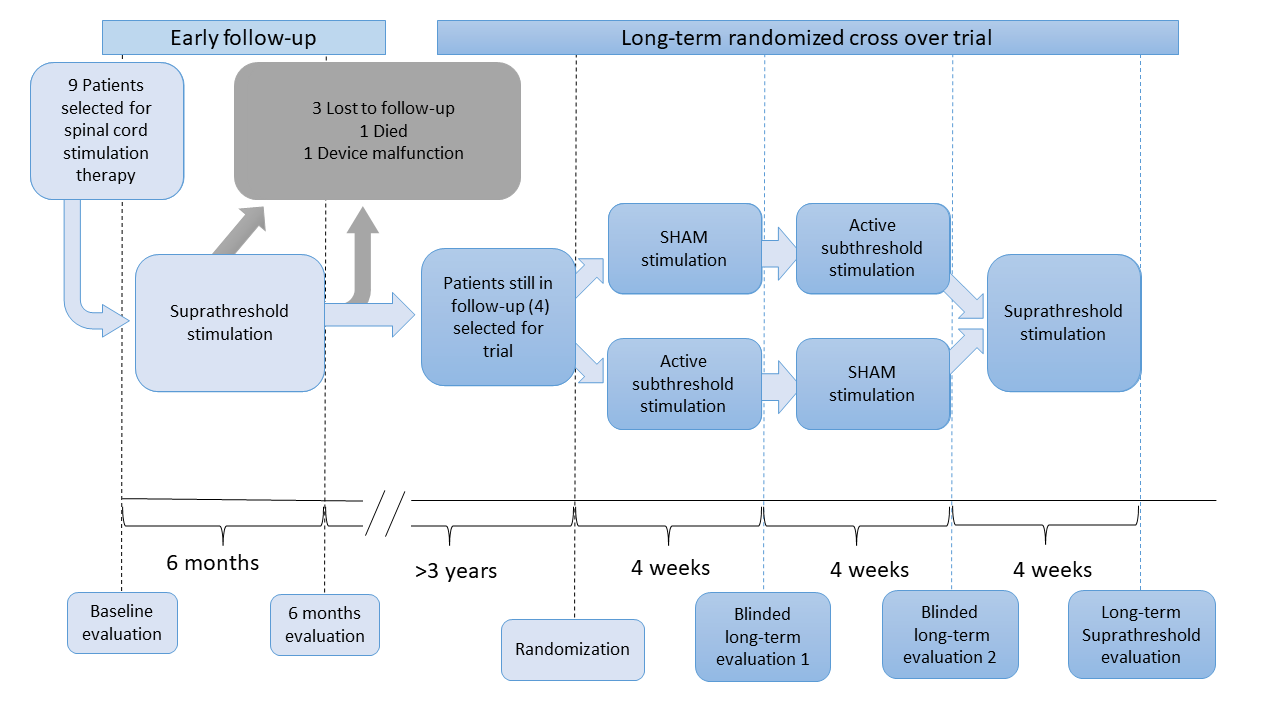


Supplementary table 1. Patient characteristics

| Characteristics | Patient 1 | Patient 2 | Patient 3 | Patient 4 |
| --- | --- | --- | --- | --- |
| Age at last evaluation and gender | 62F | 73M | 56M | 65 M |
| PD onset and duration | 40 (22) | 45 (28) | 44 (12) | 45 (20) |
| Years with DBS at last evaluation and implant site | 10, STN | 11, STN | 6, STN | No DBS |
| Years with SCS therapy at last evaluation | 7 years | 3 years | 3 years | 3 years |
| SCS indication | Refractory gait complaint | Refractory gait Freezing of Gait | Refractory pain and gait complaint | Refractory gait complaint |
| Mini Mental State Evaluation at last evaluation | Not performed – severe dysarthria. | 12 | 29 | 30 |
| Comorbidities and other important symptoms | Severe dysarthria, Dyskinesias | Dementia, prostatic hyperplasia | none | Chronic obstructive pulmonary disease, hypertension |
| LEDD pre SCS, at 6 months and at last evaluation. | 1200 / 1200 / 850 | 2128 / 2128 / 1800 | 650 / 650 / 750 | 1600 / 1800 / 2400 |
| Pain complaint at surgery | No | No | Hip, moderate | No |
| Pain complaint at last evaluation | Lower limbs, moderate | No | Hip, moderate | No |
| Hoehn Yahr scale at last evaluation | 4 | 4 | 2.5 | 2.5 |
| Patient global impression of change from SCS therapy before randomization | Much improved | No change | Minimally worse | Much improved |
| Compliance to physiotherapy and exercises | Compliant | Noncompliant | Noncompliant | Compliant |

PD Parkinson’s Disease. DBS Deep Brain Stimulation. STN Subthalamic nucleus. SCS spinal cord stimulation. LEDD Levodopa Equivalent Daily Dose.

Supplementary table 2. Outcome table.

|  | Condition | TUG (s) | FOG Q | NFOGQ | PDQ 39 | PDQ 39 mobility | V A S | F E S | MDS UPDRS part III | SCS settings amplitude / frequency / pulse width |
| --- | --- | --- | --- | --- | --- | --- | --- | --- | --- | --- |
| Patient one | Before SCS | 69 off med | 18 |  | 55.7% | 70% | 0 |  | 29 |  |
|  | 6 m ON SCS | 20.6 off med  11.6 on med | 8 |  | 33.3% | 27.5% | 0 |  | 20 | 1.1V/300Hz/90 µs |
|  | OFF long-term (blind)* | 19.7 | 15 | 25 | 52.4% | 92.5% | 6 | 59 | 61 | off |
|  | Low long-term (blind)* | 19.9 | 15 | 22 | 46.9% | 77.5% | 5 | 56 | 63 | 0.7V/300Hz/90 µs |
|  | ON SCS long term | 21.2 | 14 | 18 | 46,1% | 65% | 4 | 52 | 60 | 1.2V/300Hz/90 µs |
| Patient two | Before SCS | 15 |  | 25 | 70.5% |  | 0 |  | 44 |  |
|  | 6 m On/Off SCS | 13/ 72 |  | 26/ 27 | 59.6%** |  | 0/0 |  | 46** | 1.3V/130Hz/330 µs / off |
|  | OFF long-term (blind)* | 42.2 | 15 | 24 | 66.2% | 92.5% | 0 | 36 | 55 | off |
|  | Low long-term (blind)* | 22.1 | 14 | 25 | 64.45% | 95% | 0 | 39 | 53 | 1.0V/130Hz/330 µs |
|  | ON SCS long-term | 109 | 16 | 26 | 68.6% | 97.5% | 0 | 55 | 53 | 1.4V/130Hz/330 µs |
| Patient Three | Before SCS | 25.9 |  | 14 | 34.5% |  | 8 |  | 26 |  |
|  | 6 m ON/ Off stim | 17/ 19 |  | 12/12 | 25.6%** |  | 6/7 |  | 25** | 2.0V/170Hz/90 µs / off |
|  | OFF long-term (blind)* | 24.1 | 20 | 28 | 39.2% | 85.5% | 8 | 51 | 37 | off |
|  | Low long-term (blind)* | 21 | 17 | 28 | 35.3% | 70% | 5 | 54 | 32 | 1.3V/60Hz/300 µs |
|  | ON SCS long-term | 18.8 | 19 | 28 | 35.5% | 65% | 7 | 51 | 38 | 2.0V/60Hz/300 µs |
| Patient Four jose osni | Before SCS | 31 |  | 26 | 60.1% | 75% | 0 | 42 | 38 |  |
|  | 6 m ON/ Off stim | 11.8/13.3 |  | 28/26 | 62.17%/50% | 87.5%/80% | 0 | 47/58 | 29/29 | 5mA/500Hz/210 µs/ off |
|  | OFF long-term (blind)* | 12.8 | 14 | 23 | 57.50% | 50% | 0 | 53 | 45 | off |
|  | Low long-term (blind)* | 14.0 | 14 | 25 | 80% | 60.25% | 1 | 53 | 50 | 3.8mA/300Hz/400 µs |
|  | ON SCS long-term | 50.3 | 14 | 26 | 85% | 69.23% | 0 | 57 | 51 | 4.4ma/300Hz/400 µs |

Supplementary Table 2. Outcome measures at baseline, early follow up and double blinded evaluation * Long term OFF, low and cycling (data not in table) SCS evaluations were performed in a double blinded, randomized crossover manner using paraesthesia free subthreshold stimulation. All evaluations were performed on ON medication status except where specified for patient one. **Data available for ON SCS condition only. TUG. Timed up and go. FOGQ. Freezing of gait questionnaire. NFOG new freezing of gait questionnaire. FES fall efficiency scale. VAS visual analog scale. SCS spinal cord stimulation.

Supplementary file 4. Patient description:

Patient one, a 62-year-old female diagnosed with PD at 40 years, underwent unilateral pallidotomy in 2005 and bilateral STN DBS in 2010 due to wearing off and dyskinesias, and finally SCS device implant in 2014 due to gait complaints and FoG (Freezing of gait). She gradually developed dysartrophonia and postural instability, currently suffering approximately one fall per month and relying on single support (cane) for walking long distances. Since SCS implant, she underwent regular physical therapy sessions. Initially reporting no pain, she developed moderate bilateral knee pain in the last two years. She reported satisfaction with SCS.

Patient two is a 73-year-old man, with Parkinson onset at 45 years. He underwent unilateral pallidotomy in 2009, and bilateral STN DBS in 2010 due to motor fluctuations, and in 2018 SCS was implanted for FoG noticeable since 2015. He has had weekly falls since 2016, becoming dependent on unilateral support in the same year, and current presents with moderate dysartrophonia and frequent visual hallucinations. He reported no perceived benefit from SCS on long term evaluation. After the randomized evaluations and before the suprathreshold evaluation he was hospitalized due to pyelonephritis and discharged with a long-term urinary catheter, but could be reassessed after a four month delay.

Patient three is a 56-year-old male, with disease onset at 46 years. He received bilateral STN DBS in 2015, and SCS in October 2018 for pain and gait complaints. He suffers from important hip arthrosis resulting in moderate and constant pain since his mid-30s. He is currently not adherent to physical therapy. He relies on single support (cane) for walking long distances but usually walks unassisted. He reported nuisance with paresthesia from SCS and was dissatisfied with therapy.

Patient four is a 65-year-old male, the only patient without DBS. He received SCS for refractory gait complaints, in particular FoG, in January 2020. He additionally has mild chronic obstructive pulmonary disease and hypertension with no repercussion on functionality or well-being. He considers his gait to be much better than before SCS and is satisfied with therapy. He is adherent to physical therapy.
